# Supplementary material for: Asynchronous magnetic resonance elastography: Shear wave speed reconstruction using noise correlation of incoherent waves
Source: Magn Reson Med. 2022 Oct 27;89(3):990–1001. doi: 10.1002/mrm.29502 (PMC9792433; doi:10.1002/mrm.29502)
Supplement: Supplementary file 1 — DATA S1 MATLAB code used to generate the numerical simulations of Figure 1 [file MRM-89-990-s002.zip › k-Wave/helpfiles/example_tvsp_slit_diffraction.html]

Diffraction Through A Slit Example (k-Wave)


# Diffraction Through A Slit Example

This example illustrates the diffraction of a plane acoustic wave through a slit. It builds on the Monopole Point Source In A Homogeneous Propagation Medium and Simulating Transducer Field Patterns examples.

- Open the file in the MATLAB Editor
- Run the file in MATLAB

## Contents

- Defining the medium properties
- Defining the reference sound speed
- Running the simulation
- Diffraction through a double slit

## Defining the medium properties

The current version of k-Wave cannot explicitly enforce impedance boundary conditions. However, rigid boundaries can still be modelled by assigning a boundary with a large sound speed and density compared to the rest of the propagation medium. As this impedance difference is increased, the behaviour of the interface will approach that of a rigid boundary. Here, a diffraction slit is created by defining a thin layer with a significantly different sound speed and density to the background medium such that most of the incident wave is reflected. (Note, using `barrier_scale = 20`, some of the incident wave will still be transmitted through the barrier causing additional interference fringes. These can be reduced by increasing the barrier scale.)

```
% define the ratio between the barrier and background sound speed and density
barrier_scale = 20;

% create a mask of a barrier with a slit
slit_thickness = 2;                     % [grid points]
slit_width = 10;                        % [grid points]
slit_x_pos = Nx - Nx/4;                 % [grid points]
slit_offset = Ny/2 - slit_width/2 - 1;  % [grid points]
slit_mask = zeros(Nx, Ny);
slit_mask(slit_x_pos:slit_x_pos + slit_thickness, 1:1 + slit_offset) = 1;
slit_mask(slit_x_pos:slit_x_pos + slit_thickness, end - slit_offset:end) = 1;

% define the source wavelength to be the same as the slit size
source_wavelength = slit_width * dx;    % [m]

% assign the slit to the properties of the propagation medium
medium.sound_speed = c0 * ones(Nx, Ny);
medium.density = rho0 * ones(Nx, Ny);
medium.sound_speed(slit_mask == 1) = barrier_scale * c0;
medium.density(slit_mask == 1) = barrier_scale * rho0;
```

## Defining the reference sound speed

For a homogeneous medium, the simulations in k-Wave are unconditionally stable and free from numerical dispersion (this is a type of numerical error where the sound speed incorrectly depends on frequency which distorts the shape of the propagating waves). When the medium is heterogeneous, the calculation is only exact in regions of the domain where the sound speed matches a scalar reference sound speed used in the model. By default, k-Wave sets the reference sound speed to the maximum sound speed anywhere in the domain. This means the simulation will be unconditionally stable (it won't "blow up"), but won't prevent numerical dispersion in other parts of the domain where the sound speed is lower. In this example, the maximum sound speed is much greater than the sound speed in the background medium, and thus numerical dispersion can arise. To counteract this, it is possible to explicitly specify the value for the reference sound speed (assigned to `medium.sound_speed_ref`). However, when the reference sound speed is less than the maximum, the simulation is no longer unconditionally stable, and the time step must be chosen to be less than the stability criterion. Here, the sound speed is set to be 5% less than the maximum stable time step. A more in-depth discussion of this issue is given in the k-Wave manual.

```
% assign the reference sound speed to the background medium
medium.sound_speed_ref = c0;

% find the time step at the stability limit
c_ref = medium.sound_speed_ref;
c_max = barrier_scale * c0;
k_max = max(kgrid.k(:));
dt_limit = 2 / (c_ref * k_max) * asin(c_ref / c_max);

% create the time array, with the time step just below the stability limit
dt = 0.95 * dt_limit;   % [s]
t_end = 40e-6;          % [s]
kgrid.setTime(round(t_end / dt), dt);
```

## Running the simulation

In the first example (set `example_number = 1` within the example m-file), a sinusoidal plane wave source is created with a wavelength equal to the slit width. The source is defined as a velocity source in the x-direction extending the full width of the grid (including within the PML). This creates a plane wave source that isn't affect by the PML in the lateral direction. A visualisation of the barrier is also produced by assigning the `slit_mask` created above to the optional input parameter `'DisplayMask'`. The size of the perfectly matched layer is explicitly defined (see Controlling The Absorbing Boundary Layer Example), the simulation is run in `single` precision to reduce the computation time (see Optimising k-Wave Performance Example), and the final pressure and velocity fields are returned by `sensor.record` to `{'p_final', 'u_final'}` (see Recording The Particle Velocity Example).

```
% set the input options
input_args = {...
    'PMLSize', pml_size, ...
    'PlotPML', false, ...
    'DisplayMask', slit_mask, ...
    'DataCast', 'single', ...
    };

% run the simulation
sensor_data = kspaceFirstOrder2D(kgrid, medium, source, sensor, input_args{:});
```

A visualisation of the pressure field is given below. The wave field on the far side of the slit appears reasonably omni-directional.

In the second example (set `example_number = 2` within the example m-file), the slit size is increased and the wavelength is reduced to be a quarter of the slit width. A visualisation of the pressure field is given below. In this case, the directionality of the wavefield is increased, and interference fringes are visible.


## Diffraction through a double slit

In the third example (set `example_number = 3` within the example m-file), a double slit is used and the wavelength set equal to the slit width. The final velocity fields are also displayed.


Note, the pictures shown here were computed using `barrier_scale = 50` and `scale = 2` within the example m-file.
